# Microbial Community Profiling in Intensive Care Units Expose Limitations in Current Sanitary Standards

**DOI:** 10.3389/fpubh.2019.00240

**Published:** 2019-08-28

**Authors:** Lucas Ferreira Ribeiro, Erica M. Lopes, Luciano T. Kishi, Liliane Fraga Costa Ribeiro, Mayra Gonçalves Menegueti, Gilberto Gambero Gaspar, Rafael Silva-Rocha, María-Eugenia Guazzaroni

**Affiliations:** ^1^Department of Biology, FFCLRP -University of São Paulo, Ribeirao Preto, Brazil; ^2^Department of Cellular and Molecular Biology, FMRP -University of São Paulo, Ribeirao Preto, Brazil; ^3^National Laboratory of Scientific Computing, Petrópolis, Brazil; ^4^Department of Biochemistry and Immunology, FMRP -University of São Paulo, Ribeirao Preto, Brazil; ^5^Infection Control Service, The Medical School Clinics Hospital, University of São Paulo, Ribeirao Preto, Brazil

**Keywords:** ICU cleaning, intensive care unit, healthcare-associated infections, NICU biomarkers, cross-contamination, polyhexamethylene biguanide

## Abstract

Hospital-associated infections (HAIs) are a leading cause of morbidity and mortality in intensive care units (ICUs) and neonatal intensive care units (NICUs). Organisms causing these infections are often present on surfaces around the patient. Given that microbiota may vary across different ICUs, the HAI-related microbial signatures within these units remain underexplored. In this study, we use deep-sequencing analyses to explore and compare the structure of bacterial communities at inanimate surfaces of the ICU and NICU wards of The Medical School Clinics Hospital (Brazil). The data revealed that NICU presents higher biodiversity than ICU and surfaces closest to the patient showed a peculiar microbiota, distinguishing one unit from the other. Several facultative anaerobes or obligate anaerobes HAI-related genera were classified as biomarkers for the NICU, whereas *Pseudomonas* was the main biomarker for ICU. Correlation analyses revealed a distinct pattern of microbe-microbe interactions for each unit, including bacteria able to form multi-genera biofilms. Furthermore, we evaluated the effect of concurrent cleaning over the ICU bacterial community. The results showed that, although some bacterial populations decreased after cleaning, various HAI-related genera were quite stable following sanitization, suggesting being well-adapted to the ICU environment. Overall, these results enabled identification of discrete ICU and NICU reservoirs of potentially pathogenic bacteria and provided evidence for the presence of a set of biomarkers genera that distinguish these units. Moreover, the study exposed the inconsistencies of the routine cleaning to minimize HAI-related genera contamination.

## Introduction

Microbiome refers to the microbial community, and their respective genomes, associated with a particular habitat, including natural or built environments (1). Natural ecosystems have been well-explored; however, not much is known about indoor microbiomes—offices, houses, buildings, hospitals, etc. –where the majority of our life is spent and can have a severe impact on human health. Unlike most indoor environments, intensive care units (ICUs), or neonatal intensive care unit (NICUs) in hospitals are routinely monitored by standard cultivation techniques (2, 3). Nonetheless, conventional cultivation techniques can identify only a tiny proportion of the total bacteria (3, 4). Oberauner et al. (3) reported that only 2.5% of the overall bacterial diversity were identified in an ICU microbiome using culture-dependent methods. Culture-independent methods such as next-generation sequencing (NGS) technologies have a tremendous effect on profiling microbiomes. Phylogenetic analyses based on 16S gene diversity have been fundamental to uncover (N)ICU bacterial varieties in depth and at high resolution in space and time, and it can contribute to improving hospital safety.

In (N)ICUs, even after adopting strict sanitation protocols, many patients are infected with healthcare-associated infections (HAIs), also known as nosocomial infections, a significant public health problem around the world (5–8). HAIs include diseases that can be associated with surfaces and devices present in hospitals and can spread through health care staff, contaminated surfaces or air droplets (8). These infections are more frequent in UTIs where outbreaks often originate (9). HAIs increase deaths (morbidity and mortality), antimicrobial resistance, prolong the duration of hospital stays, and consequentially healthcare costs^1^. The National Healthcare Safety Network of the Centers for Disease Control and Prevention (CDC) has estimated 687,000 HAIs in U.S. acute care hospitals causing 72,000 deaths, and costs estimated to $97–147 billion annually (10)^2^. The most common pathogen causing HAIs are *Clostridium difficile* and “ESKAPE” bacteria (*Enterococcus spp*., *Staphylococcus aureus, Klebsiella spp*., *Acinetobacter spp*., *Pseudomonas aeruginosa*, and Enterobacteriaceae) (10, 11). Many of these bacteria exhibit antimicrobial resistance and can cause infections of the bloodstream, urinary tract, severe pneumonia, and surgical site infection (9, 12).

Hospital surfaces remain neglected reservoirs for HAI-related bacteria, and strict cleaning protocols have been used as the primary procedure to reduce the risks. Non-etheless, the efficiency of cleaning protocols, usually, has been investigated by culture-dependent routine techniques. Here, using NGS methodology, we analyzed the differences, and similarities between the structure of bacterial communities from the ICU and NICU surfaces of The Medical School Clinics Hospital (Ribeirão Preto, Brazil), one of the biggest hospitals in Latin America, and which has more than 35,000 total hospitalizations per year, and supports a population of 4 million people (https://site.hcrp.usp.br/). We hypothesized that the microbiota “signature” would vary significantly between ICU and NICU. A better understanding of these spatial biomarkers may offer opportunities for tracking the spreading of a specific microbial taxon through the hospital building. Furthermore, we tested the impact of the standard cleaning procedure established on the hospital on ICU microbiota, paying particular attention to genera associated with nosocomial infections.

## Materials and Methods

### Sample Collection and DNA Extraction

**Figure 1:**
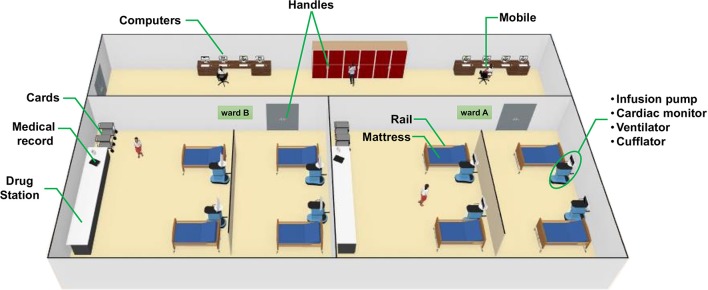
3D-rendered-model showing each sampling site of the (Neonatal) intensive care unit [(N)ICU]. ICU and NICU are on different floors at the hospital but have a similar arrangement of wards and devices in general. A detailed explanation of each sample is shown in Table 1.

A total of 158 samples were collected from the ICU and NICU at The Medical School Clinics Hospital (Ribeirão Preto, Brazil) by a single investigator from September to October 2018. The intensive care units contained two wards with four beds each, where critically ill patients from all medical specialties are treated. Samples from NICU were collected only before the concurrent cleaning, while from ICU samples were collected either before or immediately after cleaning. During sampling, all employees and devices of the ICU/NICU were in full operation. Boxes with patients lying down were swabbed on the surfaces of mattress, bed rail, monitors, infusion pumps, ventilator, and cufflator (when present). In common areas of the ICU/NICU, computer keyboard and mouse, doors handle, hospital cards, medical records, drug station, and nurse's mobiles were also swabbed. All sampling locations and their characteristics are given in Figure 1 and Table 1. The following code was used to name the samples: Samples-Unit (ICU or NICU) ward (a, b, or ab) A (after cleaning), e.g., Monitors-ICUaA Samples were collected using sterile swabs (Absorve®, Jiangsu, China) premoistened with sterile Amies media (13). The swabs were streaked across a 400 cm^2^ area in four different directions with firm movements for 2 min; swabs were rotated to ensure full contact of all parts of the swab tip and the surface. After a surface was sampled, the swab was immediately placed into sterile 15 ml Falcon tubes containing 1 mL of sterile Amies media and stored in a 4°C cooler until returning to the laboratory. In the laboratory, due to extremely low biomass, samples from a similar source and the same ward were pooled together –, e.g., four monitors from NICU ward A is a pool, and four monitors from NICU ward B another pool–generating 43 pooled samples. Then, the samples were concentrated to 500 μL by centrifugation (10,000 g/20 min), and DNA was extracted using the MoBio Powersoil DNA isolation kit, then stored in a −80°C freezer until further processing.

**Table 1:** Essential characteristics and localization of the sequenced samples.

**Sample ID**	**Sample source**	**Care unit**	**Ward**	**Cleaning**
Pumps-ICUa	Pump	ICU	A	–
Mattresses-ICUa	Mattress	ICU	A	–
Rails-ICUa	Rail	ICU	A	–
Monitors-ICUa	Monitor	ICU	A	–
Ventilators-ICUa	Ventilator	ICU	A	–
Pumps-ICUb	Pump	ICU	B	–
Mattresses-ICUb	Mattress	ICU	B	–
Rails-ICUb	Rail	ICU	B	–
Monitors-ICUb	Monitor	ICU	B	–
Ventilators-ICUb	Ventilator	ICU	B	–
MedicalRecords-ICUab	Medical record	ICU	AB	–
Cards-ICUab	Card	ICU	AB	–
Mobiles-ICUab	Mobiles	ICU	AB	–
Handles-ICUab	Handle	ICU	AB	–
DrugStations-ICUab	Drug station	ICU	AB	–
Computers-ICUab	Computer	ICU	AB	–
Pumps-ICUaA	Pump	ICU	A	+
Mattresses-ICUaA	Mattress	ICU	A	+
Rails-ICUaA	Rail	ICU	A	+
Monitors-ICUaA	Monitor	ICU	A	+
Ventilators-ICUaA	Ventilator	ICU	A	+
Pumps-ICUbA	Pump	ICU	B	+
Mattresses-ICUbA	Mattress	ICU	B	+
Rails-ICUbA	Rail	ICU	B	+
Monitors-ICUbA	Monitor	ICU	B	+
Ventilators-ICUbA	Ventilator	ICU	B	+
Cufflators-ICUabA	Cufflator	ICU	AB	+
Pumps-NICUa	Pump	NICU	A	–
Mattresses-NICUa	Mattress	NICU	A	–
Rails-NICUa	Rail	NICU	A	–
Monitors-NICUa	Monitor	NICU	A	–
Ventilators-NICUa	Ventilator	NICU	A	–
Pumps-NICUb	Pump	NICU	B	–
Mattresses-NICUb	Mattress	NICU	B	–
Rails-NICUb	Rail	NICU	B	–
Monitors-NICUb	Monitor	NICU	B	–
Ventilators-NICUb	Ventilator	NICU	B	–
Mobiles-NICUab	Mobiles	NICU	AB	–
Cards-NICUab	Card	NICU	AB	–
Handles-NICUab	Handle	NICU	AB	–
MedicalRecords-NICUab	Medical record	NICU	AB	–
DrugStations-NICUab	Drug station	NICU	AB	–
Computers-NICUab	Computer	NICU	AB	–

### Concurrent Cleaning Procedures in the ICU

At the beginning of each 24 h shift, a registered nurse washed his or her hands, put on non-sterile gloves, and wiped Boxes surfaces (mattress, bed rail, computer touch screens, monitors, infusion pumps, ventilator, and cufflator) with 1% polyhexamethylene biguanide (PHMB) solution on a soft wipe.

### Sequencing and Diversity Analysis

The DNA concentrations were measured fluorometrically (Qubit® 3.0, kit Qubit® dsDNA Broad Range Assay Kit, Life Technologies, Carlsbad, CA, USA). DNA integrity was determined by agarose gel electrophoresis using a 0.8% (w/v) gel, and subsequent staining with SYBR Safe DNA Gel Stains (Invitrogen, Carlsbad, CA, USA). A PCR was employed to amplify the V4 regions of the 16S ribosomal RNA gene 16S rRNA for bacteria (14). Each PCR reaction mixture contained 20 ng of metagenomic DNA, 10 μM of each forward and reverse primers, 1.25 mM of magnesium chloride, 200 μM of dNTP mix (Invitrogen, Carlsbad, CA, USA), 1.0 U Platinum Taq DNA polymerase high fidelity (Invitrogen, Carlsbad, CA, USA), high fidelity PCR buffer [1X], and milli-Q water. Reactions were held at 95°C for 3 min, with amplification proceeding for 30 cycles at 95°C for 30 s, 53.8°C for 30 s, and 72°C for 45 s; a final extension of 10 min at 72°C was added to ensure complete amplification. The expected fragment length of PCR products was verified by agarose gel (1%) electrophoresis, and the amplicon size was estimated by comparison with a 1 kb plus DNA ladder (1 kb plus DNA ladder, Invitrogen, Carlsbad, CA, USA). The PCR fragments were purified using the Zymoclean™ Gel DNA Recovery kit following the manufacturer's instructions. Sequencing was performed using the Miseq Reagent kit v3 2 × 300 bp.

All sequence data were processed, removing adapters using Scythe 0.991 (https://github.com/vsbuffalo/scythe) and Cutadapt 1.7.1 (15). Sequence trimming was carried out by selecting sequences over 200 bp in length with an average quality score higher than 20 based on Phred quality, and duplicate reads were removed using the Prinseq program (16). The QIIME software package version 1.9.1 was used to filter reads and determine Operational Taxonomic Units (OTUs) as described in Caporaso et al. (14). The Usearch algorithm was used to cluster the reads OTUs with a 97% cutoff, and to assign taxonomy using the Ribosomal Database Project (RDPII) version 10 (17). Bacterial sequences were de-noised, and suspected chimeras were removed using the OTU pipe function within QIIME. Sequence data were summarized at the phylum, class, and family levels; Also, Alpha_diversity.py in QIIME was used to calculate ACE, Chao1, Shannon, and Simpson indices. Principal coordinate analyses (PCoA) were conducted to evaluate differences in community structure among experimental groups (β-diversity).

For further statistical analysis and visualization, OTU table with taxa in plain format and metadata file were uploaded to the MicrobiomeAnalyst tool (available at http://www.microbiomeanalyst.ca) (18). Shallow abundant features were filtered using options: minimum count 4, low-count filter based on 20% prevalence in samples. For comparative analyses, a low variance filter was applied based on Inter-quantile range and removing the 10% lowest features. Data were rarefied to the minimum library size and normalized using total sum scaling (TSS) before any statistical comparisons (19).

## Results and Discussion

### Microbial Profiling of ICU and NICU Samples Using V4 16 rRNA Sequencing

In order to compare the microbial community of the ICU and NICU from a clinical hospital in Brazil, we use NGS targeting V4 hypervariable regions within microbial 16S rRNA genes (14). The intensive care units contained two wards with four beds each (Figure 1), where critically ill patients were present. Samples were collected from boxes areas (mattresses, bed rails, monitors, infusion pumps, ventilators, and cufflator), during the patients' hospitalization; and also from common areas (computers-keyboard and mouse, doors handle, hospital cards, medical records, drug stations, and nurse's mobiles). Furthermore, to address the question of how concurrent cleaning impacts the microbial ecosystem of an ICU, samples were collected either before or immediately after cleaning.

A total of ~1.7 million sequences corresponding to 4.94 Gbp of data from 44 samples were generated. The average number of read counts per sample was 34.621, ranging from 33.708 to 34.739. Thus, the data counts were normalized to 33.708 reads. After trimming, the final number of operational taxonomic unit (OTU) consisted of 2054, 1586, OTUs for NICU, and ICU, respectively. Rarefaction curves (Figure S1) based on the number of OTUs observed were comparably close to asymptotic for all samples. The cut-off was set to 10,000 sequences per sample whereby the rarefaction curves of all samples reached saturation, indicating the availability of enough covering to represent and compare the microbiome community present within the samples. Chimera and singleton OTU removal was included in the data processing pipeline to prevent overestimated richness. Bellow, we presented the analysis regarding the microbial composition for each sample and the comparison between the different areas analyzed.

### Comparative Assessment Between ICU and NICU Microbiota

**Figure 2:**
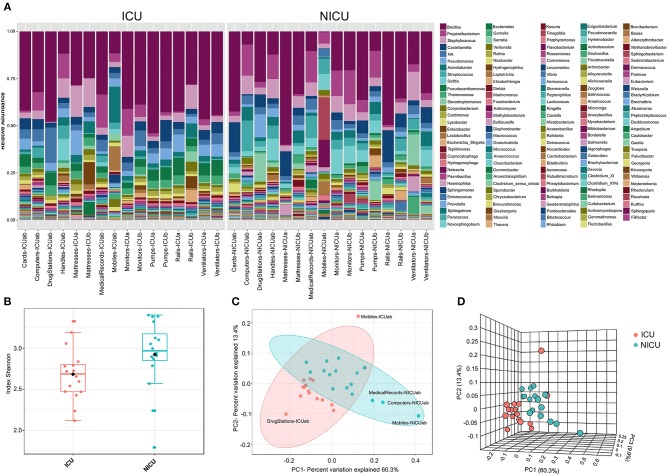
The ICU and NICU bacteria microbiota profile. **(A)** The relative abundance of bacterial genera within the top 379 OTUs among the two units. Colors correspond to the bacterial genera in the legend. Rectangles represent specific genera organized in order of abundance. Sequencing results are presented for each sample clustered using Usearch algorithm with a 97% cutoff. NA (Not_Assigned) represents sequences reads that were not assigning an accurate taxonomic label at the genus level but assigned at the higher taxonomic level. **(B)** Alpha diversity at OTU level at ICU (red, *n* = 16), and NICU (cyan, *n* = 16) calculated using Shannon index (Kruskal-Wallis test, *p* < 0.05). For each box plot herein forward, the line within the box and the black diamond represent the median and mean, respectively. The bottom and top boundaries of each box indicate the first and third quartiles (the 25th and 75th percentiles), respectively. The whiskers represent the lowest and highest values within the 1.5 interquartile range (IQR). Two- **(C)** and three-dimensional **(D)** principal coordinate analysis (PCoA) plot based on Jensen-Shannon distances between bacterial communities associated with ICU and NICU areas (ANOSIM, *R* = 0.3066; *p* < 0.001). Samples are shown as single dots. Divergence at OTU level was computed on Total sum scaling–normalized (TSS-normalized) datasets.

Microbial profiling of the ICU and NICU allowed the identification of nine different bacterial and archaeal phyla: *Firmicutes, Proteobacteria, Actinobacteria, Bacteroidetes, Fusobacteria, Cyanobacteria, Deinococcus, Gemmatimonadetes*, and *Euryarchaeota*, while this last one was only found in NICU. *Firmicutes* and *Proteobacteria* were the most abundant phyla across all samples, composing 46 and 39% of these bacterial communities, respectively. The over-representation of these phyla agree with previous results obtained for microbial communities found in (N)ICUs inanimate surfaces (3, 20–22). The microbial communities at the genus level (Figure 2A) included sequences of 138 and 160 genera, for ICU and NICU, respectively, among which a substantial number of organisms are not culturable. For all samples, the relative abundance of Not_Assigned (NA) genera was notably moderated (up to 18%). Gram-positive bacteria were found in higher abundance in both units. Non-etheless, in terms of the number of genera, Gram-negative bacteria were more diverse. The number of strictly aerobic genera were highly represented (50%) followed by facultative anaerobe (36%) and obligatory anaerobic bacteria (14%) for both units (see details in Supplemental Material). *Bacillus, Staphylococcus*, and *Pseudomonas* were the most abundant genera (47% of the total reads) on ICU surfaces, and *Bacillus, Propionibacterium* and *Staphylococcus* predominated in NICU (40%). Recent studies in other hospitals have also identified a higher abundance of *Staphylococcus* and *Pseudomonas* in ICU (23) or *Staphylococcus* and *Propionibacterium* in NICU (21, 24). These genera, including *Bacillus*, contain many commensal species for humans, although it also covers members associated with nosocomial infections in (N)ICUs. Members from these genera are considered “survival specialists,” and can persist for months on dry surfaces (25) or associated with spore or biofilm formation (26, 27). A total of 110 OTUs were found only in ICU and 578 only in NICU, while 1,476 OTUs were shared between the units (Figure S2A).

Analysis of all samples from the care units indicated that NICU samples showed a significantly higher Shannon index—a measure of diversity—as compared to samples belonging to ICU (Kruskal-Wallis test, *p* < 0.05) (Figure 2B). However, noticeable variation was observed within the sample types (Figure S2B), and computers and doors handle from both units showed the highest diversity among all samples. A higher Shannon index for NICU agrees with the differences in the number of OTUs found in the care units. The greater diversity in NICU could be explained, in part, due to the higher transit of visitors (e.g., children's parents or relatives) compared with the more restrictive transit in ICU.

Beta diversity analysis (Figures 2C,D) of the microbiota for each care unit revealed distinct, but overlapping, profile (ANOSIM, *R* = 0.3066; *p* < 0.001). A high level of variation among some samples was observed supplemented by less pronounced but distinct variation between ICU/NICU samples closer to the patient (boxes area) (ANOSIM, *R* = 0.50756; *p* < 0.001) (Figure S2C). Samples from the common area did not show a significant difference (Figure S2D). Boxes area samples from ward A and B, belonging to the same care unit, did not show a significant difference (Figures S2E,F). This analysis suggests that ICU and NICU carry a distinct microbial diversity. Besides, it is also important to remark that more significant differences were observed in the confined area closer to the patients (boxes). These areas are selective environments, where antimicrobial therapies and stringent cleaning protocols are routinely applied.

### Identification of HAI-Related Genera in Neglected (N)ICU Surfaces

**Figure 3:**
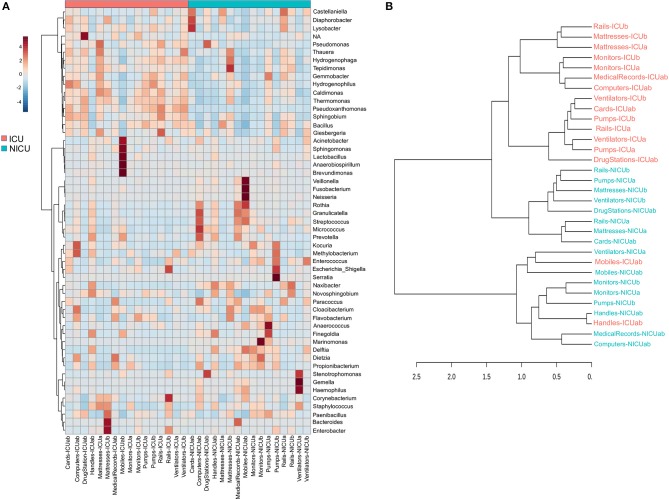
Clustering analysis of the ICU and NICU. **(A)** Heatmap, and hierarchical clustering of the main genera associated with ICU and NICU samples. The heatmap shows the relative abundance of the top 52 bacterial genera (rows) in each sample (columns). Hierarchical clustering is based on Ward Clustering algorithm and Euclidean Distance measure to generate the hierarchical tree. The color bar indicates the range of the relative abundance. **(B)** Dendrogram showing the similarities between ICU and NICU samples. The dendrogram was created using the Jaccard index as distance measure and Ward's clustering algorithm.

Evidence suggests that hospital computers (keyboard and mouse) and staff's mobiles may serve as reservoirs for bacteria associated with HAI within the healthcare environment and facilitate the cross-contamination among hospital wards (28–30). Taxonomically, ICU mobiles revealed a far greater abundance of *Acinetobacter, Sphingomonas*, and *Brevundimonas* (Figure 3A). These genera are usually found in moist environments and can show a high risk for HAI in immunocompromised patients. Besides, other genera associated with human microflora were also found in high abundances, such as *Lactobacillus* (mouth and vaginal flora) and *Anaerobiospirillum* (human, cat, and dog feces) (31). NICU mobiles showed a greater abundance of *Fusobacterium, Neisseria, Rothia, Granulicatella, and Streptococcus* (Figure 3A) that are part of the oronasopharynx or skin microflora. However, they can also be associated with severe infections in patients with a weakened immune system. Our data are consistent with previous studies that have reported that although mobiles can work as a repository to opportunistic pathogens, portions of their bacteria are also found on the human microbiome (owner's body) (32).

Computers are indispensable in contemporary hospitals, and consequently, keyboard and mouse may be contaminated with dangerous pathogenic bacteria (33, 34). Here, we found potential opportunistic genera such as *Kocuria* (present at the skin and oral flora), and *Methylobacterium* in great abundance in ICU computers whereas NICU computers were enriched with *Rothia, Granulicatella, Streptococcus, Micrococcus*, and *Prevotella* (Figure 3A). Another important, but generally neglected, potential vector of pathogens are the medical records (aka medical charts), especially those from (N)ICUs (35, 36). ICU medical records were enriched with *Dietzia* and *Flavobacterium*. NICU medical records were similar to NICU computers, except for being more abundant in *Bacteroides* (Figure 3A). Moreover, fecal indicators were detected in a high proportion of NICU medical records (Figure S3A). A hierarchical clustering analysis (Figure 3B) based on the taxonomy of the ICU and NICU samples grouped them into two major clusters. Most of the samples from the same unit were clustered together indicating their similarity. Non-etheless, the microbiota community of ICU mobiles and handles were dispersed: mobiles-ICUab clustered closely with NICU ventilators (and mobiles), while ICU handles clustered with NICU handles group. These samples belonged to a cluster that revealed an almost absent *Bacillus* and higher frequency of *Streptococcus*, among other differences (Figures 3A,B). Medical records were taxonomy similar to computers and also closer to monitors (Figure 3B). Generally, for each unit, samples from surfaces frequently touched by HCW clustered together (Figures S4A,B). These samples showed a higher abundance of skin-associated genera, which is in agreement to previous studies in (N)ICUs environments (21, 23, 37, 38). The effects of these contamination sources for the patients were not part of this study. However, based on a vast literature, it is highly recommended to sensitize healthcare staff to sanitize mobiles, hands, computers and medical records (often neglected) to prevent cross-contamination within the hospital environment.

### Identification of ICU and NICU Bacterial Biomarkers

**Figure 4:**
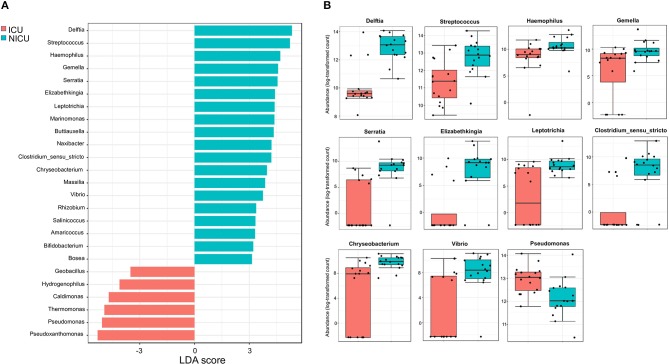
Significant differences between ICU and NICU. **(A)** Taxonomic biomarkers for ICU and NICU. Linear Discriminant Analysis (LDA) combined with Effect Size (LEfSe) indicate significant differences at the genus level that enable discrimination between the ICU and NICU samples (*p* < 0.05). Only those genera with log LDA score >3 are ultimately considered. **(B)** Boxplot of relative abundance (log scale) of the 11 HAI-related bacterial genera with significant differences between ICU (red, *n* = 16) and NICU (cyan, *n* = 16). The difference was calculated using Mann-Whitney/Kruskal-Wallis test (*p*-value and FDR < 0.05).

Across the ICU and NICU samples, different biogeographical patterns were observed for the different microbiota. LEfSe analysis was performed to identify the distinguishing genera between ICU and NICU (Figure 4A). LEfSe is a method that allows biomarker discovery most likely to explain differences between groups based on statistical significance, biological consistency, and effect relevance (39). In total, 25 genera were identified with LDA scores > 3.0. At the genus level, 11 specific biomarkers were present in NICU and 6 in ICU. All of them were both highly discriminatory and significantly different (*p*-value and FDR < 0.05) in term of abundances (Figure 4B). The HAI-related genera *Delftia, Streptococcus, Haemophilus, Gemella, Serratia, Elizabethkingia, Leptotrichia, Clostridium_sensu_stricto, Chryseobacterium*, and *Vibrio* were biomarkers for NICU. Although most of these genera can be found in the respiratory tract, mouth, vagina, and intestinal tract of healthy adults, they present a high potential for nosocomial infection in neonates. Among these genera, there is a predominance of organisms with low oxygen tolerance (facultative anaerobes or obligate anaerobes). *Pseudomonas* was identified as a biomarker for ICU. It is well-known that nosocomial infections caused by *Pseudomonas* are more often in ICUs than in other wards in the hospital (40). Except for *Streptococcus* and *Leptotrichia*, all these HAI-related genera were found mainly in surfaces closer to the patients (boxes areas). Biomarkers could be used as indicators for the microbiota status in a specific area in the hospital. Genera detected as biomarkers suggest that some bacteria can adapt extraordinarily within a particular environment.

### ICU and NICU Microbiota Have Well-defined Community-Level Structures

**Figure 5:**
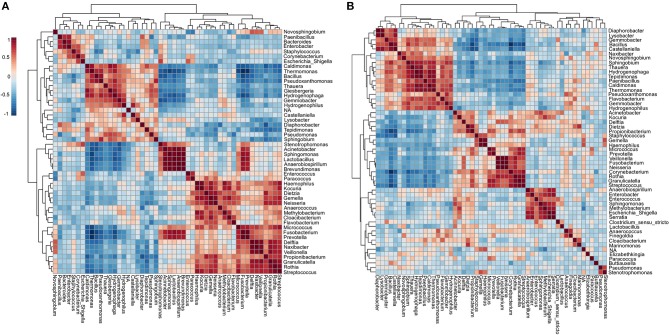
Co-occurrence and co-exclusion analysis of the bacterial genera. Heatmap showing Pearson's r correlation coefficients among the top 50 abundant bacterial genera from the **(A)** ICU and **(B)** NICU. The correlation values ranged from−1.00 (blue) to 1.00 (red). Each square represents the Pearson's r correlation coefficient between the genera of the column with that of the row. Self-self-correlations are identified in brown.

Community-level relationships among the top 50 abundant bacterial genera were investigated through Pearson's r correlation analysis (Figure 5). Microbial interaction has an essential influence on antibiotic resistance and pathogenicity. In the ICU microbiome (Figure 5A), five distinct clusters (i–v) were detected with significant positive correlations (co-occurrence). These clusters include potentially pathogenic genera such as (i) *Enterobacter, Staphylococcus, Corynebacterium*, and *Escherichia_Shigella*; (ii) Bacteria associated with outside environment (water, soil, and plants), among which *Pseudomonas*; (iii) *Stenotrophomonas, Acinetobacter, Sphingomonas*, and *Brevundimonas* (which can also cause co-infection with *Acinetobacter* spp.) (41). (iv) *Enterococcus, Haemophilus, Kocuria, Dietzia, Gemella*, and *Neisseria*; (v) *Micrococcus, Fusobacterium, Prevotella, Delftia, Veillonella, Granulicatella, Rothia*, and *Streptococcus*. Except for *Pseudomonas*, the genera *Thermomonas, Bacillus*, and *Pseudoxanthomonas* showed negative correlations with all the five clusters cited above. In the NICU (Figure 5B), we highlighted four (i–iv) clusters containing the following genera associated with nosocomial infections: (i) *Acinetobacter, Kocuria, Delftia*, and *Dietzia*; (ii) *Staphylococcus, Gemella*, and *Haemophilus*; (iii) *Fusobacterium, Neisseria, Corynebacterium, Rothia, Granulicatella*, and *Streptococcus*; (iv) *Enterobacter, Enterococcus, Sphingomonas, Escherichia*_*Shigella*, and *Serratia*. However, all these clusters revealed a strong negative correlation with *Bacillus, Sphingobium, Hydrogenophaga, Thauera, Thermomonas*, and *Gemmobacter*. It is important to note that most of these bacterial genera are known players in biofilms formation, including synergic multi-genera biofilms, on various hospital dry surfaces (23, 42). Biofilms matrix is a resistance mechanism that could stabilize a bacteria community in a selective environment such as (N)ICUs (43).

In order to verify whether the most prevalent potentially pathogenic genera identified in the ICU and NICU correlate with infected patients, 108 bacterial strains were isolated. Following standard cultivation, these strains were isolated from blood, bronchoalveolar lavage, peritoneal, cerebrospinal and ascitic fluids of hospitalized patients. All these isolates were identified, at the species level, by selective media, morphological features, and Vitek 2 rapid identification system and distributed among 12 genera. These strains comprised the genera *Klebsiella, Acinetobacter, Stenotrophomonas, Staphylococcus, Streptococcus, Pseudomonas, Enterobacter, Escherichia, Burkholderia, Cupriavidus, Morganella*, and *Ralstonia*. The most common culture-dependent isolates matched with the most abundant HAI-related genera found in the sequencing data (Figures S5A–C). This correlation shows that potentially pathogenic organisms, even when found in abundance <1% in sequencing, may be predominant in hospital infections. The majority of the isolates obtained belonged to *Staphylococcus*, which was the second more abundant Gram-positive genus found in the sequencing. *Staphylococcus* already is described as one of the most common genera found in hospitals (44).

### Investigation of ICU Microbial Community Profiling Reveals Substantial Variation on the Efficiency of the Cleaning Procedures

**Figure 6:**
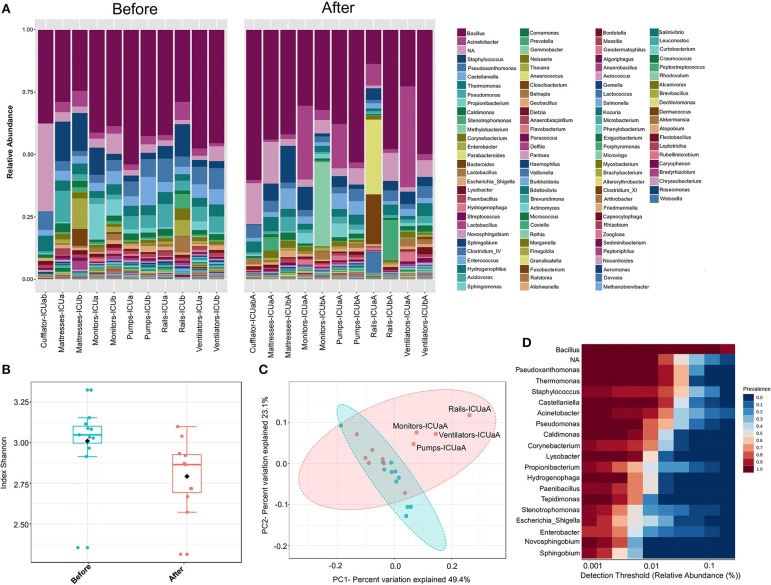
ICU bacteria microbiota profile before and after cleaning. **(A)** Relative bacterial abundance at the genus level. Sequencing results are showed for each sample surface clustered using Usearch algorithm with a 97% cutoff. Only genera with abundance > 1.0% were plotted. **(B)** Alpha diversity at OTU level, before (red, *n* = 11), and after cleaning (cyan, *n* = 11) calculated using Shannon index (Kruskal-Wallis test, *p* < 0.05). **(C)** PCoA plot based on Jensen-Shannon distances between bacterial communities associated with cleaning (ANOSIM, *R* = 0.091961; *p* = 0.039). Samples are shown as single dots. Divergence at OTU level was computed on Total sum scaling–normalized (TSS-normalized) datasets. **(D)** Core microbiome analysis based on relative abundance and sample prevalence of bacterial genus before and after cleaning.

Cleaning procedures at ICUs are an important practice to prevent HAI-related bacteria spreading (44) Although the protocols may vary between hospitals, concurrent cleaning procedures involved strict disinfection and sterilization of patient supplies and equipment during hospitalization. Here, the antimicrobial solution used for daily ICU cleaning contained the cationic polymer polyhexamethylene biguanide (PHMB). A recent model suggests that PHMB enter bacterial cells and condenses chromosomes, inhibiting cell division (45). Thus, in order to investigate how concurrent cleaning affects the ICU microbiome, samples from surfaces near patients were sequencing, and analyzed either before or immediately after cleaning. The microbial communities at the genus level included sequences of 117 and 94 genera, for before and after cleaning, respectively (Figure 6A). Seven percent of the OTUs could not be classified to genera level (NA). These unclassified groups had higher relative abundance in cufflator-ICUab (35%). Samples after cleaning showed a slight but significant decrease in the diversity (Kruskal-Wallis test, *p* < 0.05) (Figure 6B). However, noticeable variation was observed within the sample types (Figure S6). Beta diversity analysis revealed a distinct, but overlapping, profile (*R* = 0.091961; *p* < 0.05) (Figure 6C). Most of the samples from ICU ward-A after cleaning clustered separately from the rest of the surfaces. Quite remarkably, these differences in diversity after cleaning reveal that the procedure did not have the same effect on all surfaces. Although it is known that different microbiomes may exert different effects on cleaning (23) this was not the case, since no significant difference between room A and B was observed prior to cleaning. Therefore, differences in the effect of cleanliness on diversity could be explained, in part, by a lack of standardization in the protocol.

**Figure 7:**
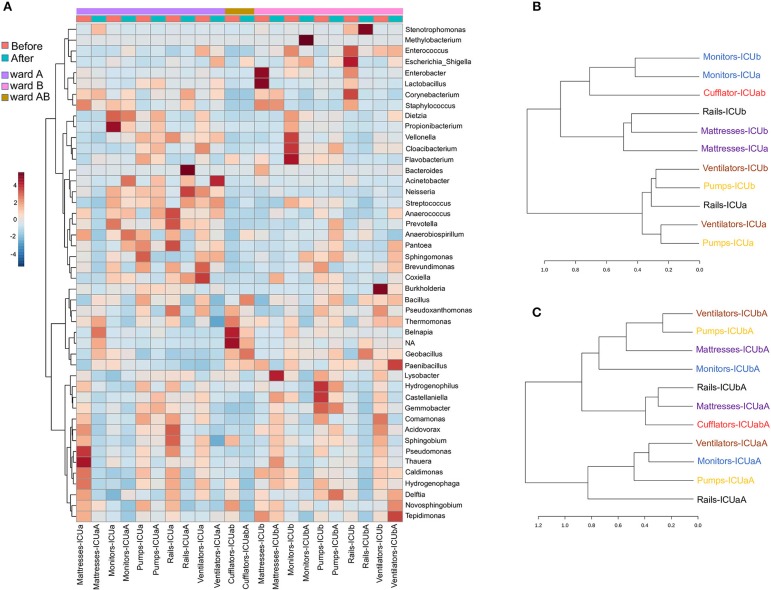
Clustering analysis of the ICU samples before and after cleaning. **(A)** Heatmap of the main genera associated with ICU samples before and after cleaning. The heatmap shows the relative abundance of top 45 bacterial genera (rows) in each sample (columns). The color bar indicates the range of the relative abundance. Dendrogram showing the similarities between samples **(B)** before and **(C)** after cleaning. The dendrogram was created using the Jaccard index as distance measure and Ward's clustering algorithm.

The samples either before or after cleaning were inhabited by high relative abundances (~65%) of *Bacillus, Pseudoxanthomonas, Thermomonas, Staphylococcus, Castellaniella*, and *Acinetobacter*. Core microbiome analysis showed that 19 genera were shared in 80% of all samples (before and after) at the minimum detection threshold of 0.001% relative abundance (Figure 6D). Most notably, the most abundant genera were also clearly most prevalent in the core microbiome before and after cleaning. Gram-positive bacteria were found in higher abundance (before−53%; after−51%, respectively), showing 45 different genera before and 30 after cleaning (33% less). Furthermore, Gram-negative bacteria revealed higher diversity, with 72 genera before and 64 after cleaning (11% less). Most of the genera absent after cleaning showed very low abundance (< 0.05%) before cleaning. The HAI-related organism *Chryseobacterium*, and *Clostridium_XI* are among the genera absent (or extremely low) after cleaning. Besides these absent genera, using the statistical parameters *p*-value and FDR < 0.05, no other analyzed genera showed a significant difference between the average abundance calculated for all samples before and after cleaning. However, the HAI-related genera *Comamonas, Pseudomonas, Enterobacter, Kocuria, Ralstonia*, and *Delfitia* showed a decrease, while *Leptotrichia, Streptococcus*, and *Acinetobacter* presented an increase on average abundance ≥2-fold after cleaning (Figure S7A). Curiously, cleaning efficiency was notably variable among the samples (Figure S7B). Previous studies have shown that even with strict cleaning procedures, HAI-related genera, such as *Staphylococcus, Klebsiella, Acinetobacter, Pseudomonas, Enterococcus, Escherichia*, and *Enterobacter*, are generally found on the surface of the ICU devices (46–50). To examine more deeply the cleaning effect among the samples, a heatmap of the top 45 genera is illustrated in Figure 7A. The cleaning efficiency was not the same through the samples and wards. Some genera showed a tendency to decrease after cleansing, such as *Enterococcus, Enterobacter, Staphylococcus, Burkholderia, Comamonas, Pseudomonas*, and *Delftia*. However, others increased in one ward and dropped in the other, such as *Corynebacterium*, and *Acinetobacter* (increased for ward-A and decreased for ward-B) or *Prevotella*, and *Novosphingobium* (decrease for ward-A and increase for ward-B).

Moreover, there were genera that revealed an tremendous increasing after cleaning in some specific surfaces, such as *Stenotrophomonas* (mattresses-ICUaA and rails-ICUbA), *Methylobacterium* (monitors-ICUbA), *Bacteroides, Neisseria*, and *Streptococcus* (rails-ICUaA), *Acinetobacter* and *Escherichia* (ventilators-ICUaA), *Dietzia* (monitors-ICUaA), *Delftia* (pumps-ICUbA), *Novosphingobium*, and *Tepidimonas* (ventilators-ICUbA). Fecal indicators were detected in higher abundance after cleaning on bed rails (mainly on Rails-ICUaA) (Figure S3A). These results reveal that cleaning was inconsistent and, in some cases, increased the abundance of specific genera. Previous studies have shown that hands are one of the primary vectors of HAI-related bacterial cross-contamination (51, 52), mainly because of the variable compliance on hands hygiene and gloves changing after touching surfaces near to the patients (53). Besides, disinfectant solutions and wipes used for hospital cleaning also can be a vital source of pathogen transfer and inconsistency in surfaces cleaning, even when standard protocols are followed (54). Furthermore, other factors to be considered is the low efficiency of PHMB-based products in relation to contaminations by wound secretions or urine containing a massive load of bacteria (55), and a possible discrepancy in the cleaning procedure performed by different nurses. Based on hierarchical clustering analysis, before cleaning (Figure 7B) most of the samples with the same functionality, but from different wards, were clustered together indicating their similarity. Non-etheless, the microbiota community after cleaning (Figure 7C) revealed a higher dispersion among the samples. We speculate that cleaning could be a way of spreading colonizing genera from one surface to another, but that over time there may be a reestablishment of the microbial community related to a specific sample.

### Cleaning Procedures Generates Substantial Rearrangements in the Community-Level Structures

**Figure 8:**
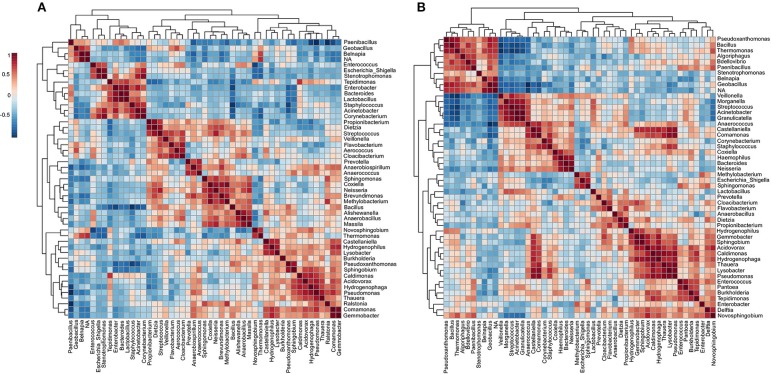
Co-occurrence and co-exclusion analysis of the bacterial genera. Heatmap showing Pearson's r correlation coefficients among the top 50 abundant bacterial genera from the **(A)** ICU before and **(B)** ICU after cleaning. The correlation values ranged from−1.00 (blue) to 1.00 (red). Each square represents the Pearson's r correlation coefficient between the genera of the column with that of the row. Self-self-correlations are identified in brown.

To investigate the changes in the microbial community structure before and after cleaning, the correlation coefficients among the top 50 genera was analyzed (Figures 8A,B). For the microbiome before cleaning, four distinct clusters (i–iv) were detected with significant positive co-occurrence (Figure 8A). These clusters include potentially pathogenic genera such as (i) *Enterococcus, Escherichia_Shigella, Stenotrophomonas, Enterobacter, Staphylococcus, Acinetobacter*, and *Corynebacterium*; (ii) *Dietzia, Streptococcus*, and *Veillonella*; (iii) *Sphingomonas, Neisseria*, and *Methylobacterium*; (iv) *Burkholderia, Pseudomonas, Ralstonia*, and *Comamonas*. The environmental genus *Belnapia* showed negative correlations with all the genera cited above.

After cleaning, six clusters (i–vi) are presented (Figure 8B) containing highlighted genera associated with nosocomial infections: (i) *Stenotrophomonas* and other environmental genera; (ii) *Veillonella, Morganella, Streptococcus, Acinetobacter, Granulicatella, Comamonas, Corynebacterium, Staphylococcus, Haemophilus*, and *Neisseria*; (iii) *Methylobacterium, Escherichia*, and *Sphingomonas*. (iv) *Prevotella* and other genera related to low oxygen tolerance or vaginal microbiome (56); (v) *Pseudomonas, Enterococcus, Pantoea*, and *Burkholderia*; (vi) *Enterobacter, Delftia*, and *Novosphingobium*. However, most of these HAI-related genera revealed a strong negative correlation with *Pseudoxanthomonas* (except *Delftia* and *Novosphingobium*). The correlation data showed a predominance of Proteobacteria among most of the clusters. Proteobacteria are predominant in the skin of the forearm (57) and are highly associated with biofilms formation on the surface of devices used in ICUs (42). Several genera relationships were quite stable to disinfection stress because it was found clustered both before and after cleaning. In all the clusters were found genera associated with species able to form biofilms. Genera associated with xenobiotic metabolism were found among the clusters i–iv, and ii–v before and after cleaning, respectively, (58, 59). After cleaning, a redistribution of some genera in new clusters was noticed. For example, a more extensive cluster involving 10 HAI-related genera (ii) was formed after cleaning, and this cluster included a mixture of several genera found in clusters i–iv before cleaning. Although this cluster analysis is useful to visualize the dynamics of microbiota with the cleaning efficiency, further studies will be required to understand the exact changes in the microbe-microbe interactions underlying the differences observed across time.

## Conclusions

The relevance of spatial composition of the microbial communities within a hospital is unclear. To our knowledge, this is the first study using deep sequencing of inanimate surfaces samples to develop a spatial assessment of the microbial community in ICU and NICU wards within the same hospital. In this comprehensive study, we observed a peculiar spatial structure between ICU and NICU microbiota in one of the largest hospitals in Brazil. The data revealed that among the samples analyzed, NICU presents higher biodiversity than in the ICU. Genera considered “survival specialists” are among the most persistent and abundant in both units. Areas closest to the patient hold more specific microbiota, distinguishing one unit from other. Most of the genera found in both units are present in the healthy human microbiome, suggesting that the most likely vectors of contamination are hospital staff and patients. Most of these genera can also be associated with nosocomial infection, especially for patients in (N)ICU. Devices commonly used, but generally neglected, such as mobile phones, computers, and medical charts are enriched with HAI-related genera (e.g., *Acinetobacter, Fusobacterium, Kocuria, Rothia*, and *Dietzia*). For the samples analyzed in the present study, some facultative anaerobes or obligate anaerobes genera were classified as biomarkers for the NICU (e.g., *Serratia* and *Clostridium*), whereas *Pseudomonas* as a biomarker for ICU. Correlation analyses revealed a distinct pattern of microbe-microbe interactions for each unit, including several bacteria able to form multi-genera biofilms. Cultivation-dependent results showed a positive correlation between the most abundant HAI-related genera identified by sequencing with infections found in the hospital. According, our data showed similarity with previous studies and can help to define soon what constitutes a “typical” microbiome in the ICU and NICU environments. The ability to identify HAI-related genera that are spatially concentrated in a hospital ward may influence the future use of improved tools and protocols for infection control.

Furthermore, we evaluated the effect of concurrent cleaning over the ICU bacterial community. Cleaning showed a slight decrease in diversity. Several genera were quite stable to disinfection, suggesting being well-adapted to the ICU environment. In general, the cleaning procedure was inconsistent. Potential influencing factors from the unsatisfactory cleaning include low efficiency of the biocide used, bacteria well-adapted to daily cleaning, disinfectant solutions and wipes contaminated, and variable compliance on hands hygiene and cleaning procedure. Therefore, this type of analysis can be used for designing better strategies for cleaning procedures.

It is also important to highlight the two major drawbacks of 16S rRNA sequencing for pathogen detection: i) the lack of taxonomic resolution at the strain-level. Thus, within all the genera found here, there are species (opportunistic) pathogenic bacteria, as well as harmless and beneficial bacteria. Shotgun sequencing would be a more accurate approach to access strain-level; however, it is very challenging to apply for low biomass samples (e.g., surface swabs), and it is particularly vulnerable to bias due to the sample size issue (60). Although our 16S study limited our ability to infer the contamination patterns of strains with specific clinical relevance, we observed an important correlation between the most abundant HAI-related genera and the isolated strains from infected patients. This correlation highlighted that potentially pathogenic genera identified by 16S sequencing may be predominant in hospital infections. (ii) another major downside of 16S rRNA sequencing is the fact that it does not test viability, the sequencing reveals DNA of dead as well as live bacteria. Nevertheless, the “dead” DNA must be considered as a biological reservoir, since most of the bacteria can incorporate and spread this DNA by horizontal gene transfer and transformation (61), contributing to boosting the bacterial virulence in the hospital.

In conclusion, due to the high impact of HAIs, there is an urgent need for the development of robust policies on microbial surveillance to help guide procedures, improving infection control. This work highlighted genera associated with nosocomial infections, identifying the most potent reservoirs of microbial transfer, and evaluated the microbiota changes related to a standardized cleaning procedure followed worldwide by hospital staff. However, further investigation is needed to clarify the microbial structure in species-level. Therefore, this study contributes to increase the knowledge about (N)ICUs microbiomes and may help to reduce health-care-associated infections, especially in developing countries.

## Data Availability

The nucleotide sequences obtained in the present study have been deposited in the GenBank database under the Accession number PRJNA541082.

## Author's Note

This manuscript has been released as a Pre-Print at bioRxiv (doi: 10.1101/633404).

## Author Contributions

LFR, RS-R, and M-EG conceived of the project and wrote the final manuscript. LFR, LCR, MM, and GG organized the sample collections. LFR conducted the nucleic acid extractions and contributed to the data analysis. EL and LK conducted the MiSeq library preparations and provided the bioinformatics support. All authors have read and approved the manuscript.

### Conflict of Interest Statement

The authors declare that the research was conducted in the absence of any commercial or financial relationships that could be construed as a potential conflict of interest. The reviewer AA declared a shared affiliation, with no collaboration, with the authors to the handling editor at the time of review.

## Footnotes

^1^CDC. Healthcare-asociated infections. Available from: https://www.cdc.gov/hai/index.html).

^2^CDC. HAI Hospital Prevalence Survey. Available from: https://www.cdc.gov/hai/data/portal/index.html).

## References

[1] Marchesi JR Ravel J The vocabulary of microbiome research: a proposal. Microbiome. (2015) 31:1–3. 10.1186/s40168-015-0094-5 PMC4520061 26229597

[2] Daneman N Stukel TA Ma X Vermeulen M Guttmann A. Reduction in clostridium difficile infection rates after mandatory hospital public reporting: findings from a longitudinal cohort study in Canada. PLoS Med. (2012) 9:e1001268. 10.1371/journal.pmed.1001268 22815656 PMC3398960

[3] Oberauner L Zachow C Lackner S Högenauer C Smolle KH Berg G. The ignored diversity: Complex bacterial communities in intensive care units revealed by 16S pyrosequencing. Sci Rep. (2013) 3:1413. 10.1038/srep01413 23475210 PMC3593336

[4] Staley JT Konopka A. Measurement of *in situ* activities of nonphotosynthetic microorganisms in aquatic and terrestrial habitats. Annu Rev Microbiol. (2003) 39:321–46. 10.1146/annurev.mi.39.100185.001541 3904603

[5] Calfee DP. Crisis in hospital-acquired, healthcare-associated infections. Annu Rev Med. (2011) 63:359–71. 10.1146/annurev-med-081210-144458 22017445

[6] Teerawattanapong N Panich P Kulpokin D Na Ranong S Kongpakwattana K Saksinanon A. A systematic review of the burden of multidrug-resistant healthcare-associated infections among intensive care unit patients in Southeast Asia: the rise of multidrug-resistant acinetobacter baumannii. Infect Control Hosp Epidemiol. (2018) 39:525–33. 10.1017/ice.2018.58 29580299

[7] Walter J Haller S Quinten C Kärki T Zacher B Eckmanns T. Healthcare-associated pneumonia in acute care hospitals in European union/European economic area countries: an analysis of data from a point prevalence survey, 2011 to 2012. Eurosurveillance. (2018) 23:1–12. 10.2807/1560-7917.ES.2018.23.32.1700843 30107871 PMC6092912

[8] WHO (2011). Report on the Burden of Endemic Health Care-Associated Infection Worldwide. Available online at: https://apps.who.int/iris/handle/10665/80135 (accessed May 05, 2019).

[9] Agaba P Tumukunde J Tindimwebwa JVB Kwizera A. Nosocomial bacterial infections and their antimicrobial susceptibility patterns among patients in Ugandan intensive care units: a cross sectional study. BMC Res Notes. (2017) 10:349. 10.1186/s13104-017-2695-5 28754148 PMC5534037

[10] Magill SS Edwards JR Bamberg W Beldavs ZG Dumyati G Kainer MA. Multistate point-prevalence survey of health care–associated infections. N Engl J Med. (2014) 370:1198–208. 10.1056/NEJMoa1306801 24670166 PMC4648343

[11] Magill SS O'Leary E Janelle SJ Thompson DL Dumyati G Nadle J. Changes in prevalence of health care–associated infections in U.S. Hospitals. N Engl J Med. (2018) 379:1732–44. 10.1056/NEJMoa1801550 30380384 PMC7978499

[12] Lax S Gilbert JA. Hospital-associated microbiota and implications for nosocomial infections. Trends Mol Med. (2015). 21:427–32. 10.1016/j.molmed.2015.03.005 25907678

[13] Amies CR. A modified formula for the preparation of Stuart's Transport Medium. Can J Public Heal. (1967) 58:296–300. 4859908

[14] Caporaso JG Lauber CL Walters WA Berg-Lyons D Lozupone CA Turnbaugh PJ. Global patterns of 16S rRNA diversity at a depth of millions of sequences per sample. Proc Natl Acad Sci. (2011) 108(Suppl 1):4516–22. 10.1073/pnas.1000080107 20534432 PMC3063599

[15] Martin M Cutadapt removes adapter sequences from high-throughput sequencing reads. EMBnet J. (2011) 17:10 10.14806/ej.17.1.200

[16] Schmieder R Edwards R. Quality control and preprocessing of metagenomic datasets. Bioinformatics. (2011) 27:863–4. 10.1093/bioinformatics/btr026 21278185 PMC3051327

[17] Cole JR Wang Q Fish JA Chai B McGarrell DM Sun Y. Ribosomal Database Project: data and tools for high throughput rRNA analysis. Nucleic Acids Res. (2014) 42:D633–42. 10.1093/nar/gkt1244 24288368 PMC3965039

[18] Dhariwal A Chong J Habib S King IL Agellon LB Xia J. MicrobiomeAnalyst: a web-based tool for comprehensive statistical, visual and meta-analysis of microbiome data. Nucleic Acids Res. (2017) 45:180–8. 10.1093/nar/gkx295 28449106 PMC5570177

[19] Paulson JN Colin Stine O Bravo HC Pop M. Differential abundance analysis for microbial marker-gene surveys. Nat Methods. (2013) 10:1200–2. 10.1038/nmeth.2658 24076764 PMC4010126

[20] Bokulich NA Mills DA Underwood MA. Surface microbes in the neonatal intensive care unit: changes with routine cleaning and over time. J Clin Microbiol. (2013) 51:2617–24. 10.1128/JCM.00898-13 23740726 PMC3719657

[21] Brooks B Firek BA Miller CS Sharon I Thomas BC Baker R. Microbes in the neonatal intensive care unit resemble those found in the gut of premature infants. Microbiome. (2014) 2:1–16. 10.1186/2049-2618-2-1 24468033 PMC4392516

[22] Poza M Gayoso C Gómez MJ Rumbo-Feal S Tomás M Aranda J. Exploring bacterial diversity in hospital environments by GS-FLX titanium pyrosequencing. PLoS ONE. (2012) 7:e44105. 10.1371/journal.pone.0044105 22952889 PMC3430676

[23] Hu H Johani K Gosbell IB Jacombs ASW Almatroudi A Whiteley GS. Intensive care unit environmental surfaces are contaminated by multidrug-resistant bacteria in biofilms: combined results of conventional culture, pyrosequencing, scanning electron microscopy, and confocal laser microscopy. J Hosp Infect. (2015) 91:35–44. 10.1016/j.jhin.2015.05.016 26187533

[24] Hewitt KM Mannino FL Gonzalez A Chase JH Caporaso JG Knight R. Bacterial diversity in two Neonatal Intensive Care Units (NICUs). PLoS ONE. (2013) 8:e54703. 10.1371/journal.pone.0054703 23372757 PMC3553055

[25] Kramer A Schwebke I Kampf G. How long do nosocomial pathogens persist on inanimate surfaces? BMC Infect Dis. (2006) 6:130. 10.1186/1471-2334-6-130 16914034 PMC1564025

[26] Achermann Y Goldstein EJC Coenye T Shirtliffa ME. Propionibacterium acnes: from commensal to opportunistic biofilm-associated implant pathogen. Clin Microbiol Rev. (2014) 27:419–40. 10.1128/CMR.00092-13 24982315 PMC4135900

[27] Majed R Faille C Kallassy M Gohar M. Bacillus cereus biofilms-same, only different. Front Microbiol. (2016) 7:1054. 10.3389/fmicb.2016.01054 27458448 PMC4935679

[28] Badr RI Badr HI Ali NM Mobile phones and nosocomial infections. Int J Infect Control. (2012) 8, 1–5. 10.3396/ijic.v8i2.014.12

[29] Brady RRW Verran J Damani NN Gibb AP. Review of mobile communication devices as potential reservoirs of nosocomial pathogens. J Hosp Infect. (2009) 71:295–300. 10.1016/j.jhin.2008.12.009 19168261

[30] Brady RR Hunt AC Visvanathan A Rodrigues MA Graham C Rae C. (2011). Mobile phone technology and hospitalized patients: a cross-sectional surveillance study of bacterial colonization, and patient opinions and behaviours. Clin Microbiol Infect. 17:830–5. 10.1111/j.1469-0691.2011.03493.x 21615607

[31] Malnick H Williams K Phil-Ebosie J Levy AS. Description of a medium for isolating *Anaerobiospirillum* spp., a possible cause of zoonotic disease, from diarrheal feces and blood of humans and use of the medium in a survey of human, canine, and feline feces. J Clin Microbiol. (1990) 28:1380–4. 2380364 10.1128/jcm.28.6.1380-1384.1990 PMC267936

[32] Meadow JF Altrichter AE Green JL. Mobile phones carry the personal microbiome of their owners. PeerJ. (2014) 2:e447. 10.7717/peerj.447 25024916 PMC4081285

[33] Bures S Fishbain JT Uyehara CFT Parker JM Berg BW Computer keyboards and faucet handles as reservoirs of nosocomial pathogens in the intensive care unit. Am J Infect Control. (2000) 6:465–71. 10.1067/mic.2000.107267 11114617

[34] Rutala WA White MS Gergen MF Weber D.(2007). Bacterial contamination of keyboards: efficacy and functional impact of disinfectants. Obstet Gynecol Surv. 103–4. 10.1097/01.ogx.0000253752.51984.e7 16622815

[35] Chen KH Chen LR Wang YK. Contamination of medical charts: an important source of potential infection in hospitals. PLoS ONE. (2014) 9:e78512. 10.1371/journal.pone.0078512 24558355 PMC3928153

[36] Teng S-O Lee W-S Ou T-Y Hsieh Y-C Lee W-C Lin Y-C. Bacterial contamination of patients' medical charts in a surgical ward and the intensive care unit: impact on nosocomial infections. J Microbiol Immunol Infect. (2009) 42:86–91. 19424563

[37] Dominguez-Bello MG Costello EK Contreras M Magris M Hidalgo G Fierer N. Delivery mode shapes the acquisition and structure of the initial microbiota across multiple body habitats in newborns. Proc Natl Acad Sci. (2010) 107:11971–5. 10.1073/pnas.1002601107 20566857 PMC2900693

[38] Handorean A Hull N Hernandez M Robertson CE Pace NR Harris JK Microbial aerosol liberation from soiled textiles isolated during routine residuals handling in a modern health care setting. Microbiome. (2015) 72:1–10. 10.1186/s40168-015-0132-3 PMC4673858 26646166

[39] Segata N Izard J Waldron L Gevers D Miropolsky L Garrett WS. Metagenomic biomarker discovery and explanation. Genome Biol. (2011) 12:R60. 10.1186/gb-2011-12-6-r60 21702898 PMC3218848

[40] Donowitz LG Wenzel RP Hoyt JW. High risk of hospital-acquired infection in the ICU patient. Crit Care Med. (1982) 10:355–7. 10.1097/00003246-198206000-00001 7075228

[41] Ryan MP Pembroke JT. *Brevundimonas* spp: Emerging global opportunistic pathogens. Virulence. (2018) 9:480–93. 10.1080/21505594.2017.1419116 29484917 PMC5955483

[42] Perez E Williams M Jacob JT Reyes MD Tejedor SC Steinberg JP. Microbial biofilms on needleless connectors for central venous catheters: comparison of standard and silver-coated devices collected from patients in an acute care hospital. J Clin Microbiol. (2014) 52:823–31. 10.1128/JCM.02220-13 24371233 PMC3957745

[43] Fux CA Costerton JW Stewart PS Stoodley P. Survival strategies of infectious biofilms. Trends Microbiol. (2005) 13:34–40. 10.1016/j.tim.2004.11.010 15639630

[44] Mora M Mahnert A Koskinen K Pausan MR Oberauner-Wappis L Krause R. Microorganisms in confined habitats: microbial monitoring and control of intensive care units, operating rooms, cleanrooms and the international space station. Front Microbiol. (2016) 7:1573. 10.3389/fmicb.2016.01573 27790191 PMC5061736

[45] Chindera K Mahato M Kumar Sharma A Horsley H Kloc-Muniak K Kamaruzzaman NF. The antimicrobial polymer PHMB enters cells and selectively condenses bacterial chromosomes. Sci Rep. (2016) 6:23121. 10.1038/srep23121 26996206 PMC4800398

[46] Livornese LL Dias S Samel C Romanowski B Taylor S May P. Hospital-acquired infection with vancomycin-resistant *Enterococcus faecium* transmitted by electronic thermometers. Ann Intern Med. (1992) 117:112–6. 10.7326/0003-4819-117-2-112 1605425

[47] Marinella MA. The stethoscope. A potential source of nosocomial infection? Arch Intern Med. (2003) 157:786–90. 10.1001/archinte.157.7.786 9125011

[48] Myers MG. Longitudinal evaluation of neonatal nosocomial infections: association of infection with a blood pressure cuff. Pediatrics. (1978) 61:42–5. 263872

[49] Safdar N Drayton J Dern J Warrack S Duster M Schmitz M Telemetry leads harbor nosocomial pathogens. Int J Infect Control. (2012) 8:1–3. 10.3396/ijic.v8i2.012.12

[50] Schabrun S Chipchase L Rickard H. Are therapeutic ultrasound units a potential vector for nosocomial infection? Physiother Res Int. (2006) 11:61–71. 10.1002/pri.329 16808087

[51] Agodi A Barchitta M Cipresso R Giaquinta L Romeo MA Denaro C. Pseudomonas aeruginosa carriage, colonization, and infection in ICU patients. Intensive Care Med. (2007) 33:1155–61. 10.1007/s00134-007-0671-6 17503016

[52] Weber DJ Rutala WA Miller MB Huslage K Sickbert-Bennett E. Role of hospital surfaces in the transmission of emerging health care-associated pathogens: Norovirus, Clostridium difficile, and Acinetobacter species. Am J Infect Control. (2010) 38:25–33. 10.1016/j.ajic.2010.04.196 20569853

[53] Ali S Wilson APR. Effect of poly-hexamethylene biguanide hydrochloride (PHMB) treated non-sterile medical gloves upon the transmission of Streptococcus pyogenes, carbapenem-resistant E. *coli*, MRSA and Klebsiella pneumoniae from contact surfaces. BMC Infect Dis. (2017) 17:574. 10.1186/s12879-017-2661-9 28814284 PMC5559802

[54] Ramm L Siani H Wesgate R Maillard JY. Pathogen transfer and high variability in pathogen removal by detergent wipes. Am J Infect Control. (2015) 43:724–8. 10.1016/j.ajic.2015.03.024 25997876

[55] Hedin G Rynbäck J Loré B. Reduction of bacterial surface contamination in the hospital environment by application of a new product with persistent effect. J Hosp Infect. (2010). 75:112–5. 10.1016/j.jhin.2010.02.007 20381907

[56] Younes JA Lievens E Hummelen R van der Westen R Reid G Petrova MI.(2018). Women and their microbes: the unexpected friendship. Trends Microbiol. 26:16–32. 10.1016/j.tim.2017.07.008 28844447

[57] Grice EA Segre JA. (2011). The skin microbiome. Nat Rev Microbiol. 9:244–253. 10.1038/nrmicro2537 21407241 PMC3535073

[58] Das A Srinivasan M Ghosh TS Mande SS. Xenobiotic metabolism and gut microbiomes. PLoS ONE. (2016) 11:e0163099. 10.1371/journal.pone.0163099 27695034 PMC5047465

[59] Haiser HJ Turnbaugh PJ. (2013). Developing a metagenomic view of xenobiotic metabolism. Pharmacol Res. 69:21–31. 10.1016/j.phrs.2012.07.009 22902524 PMC3526672

[60] Salter SJ Cox MJ Turek EM Calus ST Cookson WO Moffatt MF. Reagent and laboratory contamination can critically impact sequence-based microbiome analyses. BMC Biol. (2014) 12:87. 10.1186/s12915-014-0087-z 25387460 PMC4228153

[61] Soucy SM Huang J Gogarten JP. (2015). Horizontal gene transfer: building the web of life. Nat Rev Genet. 16:472–82. 10.1038/nrg3962 26184597

