# Supplementary material for: Microbial Community Profiling in Intensive Care Units Expose Limitations in Current Sanitary Standards
Source: Front Public Health. 2019 Aug 28;7:240. doi: 10.3389/fpubh.2019.00240 (PMC6724580; doi:10.3389/fpubh.2019.00240)
Supplement: Supplementary file 1 [file Data_Sheet_1.PDF]

Supplementary Material

Figure S1

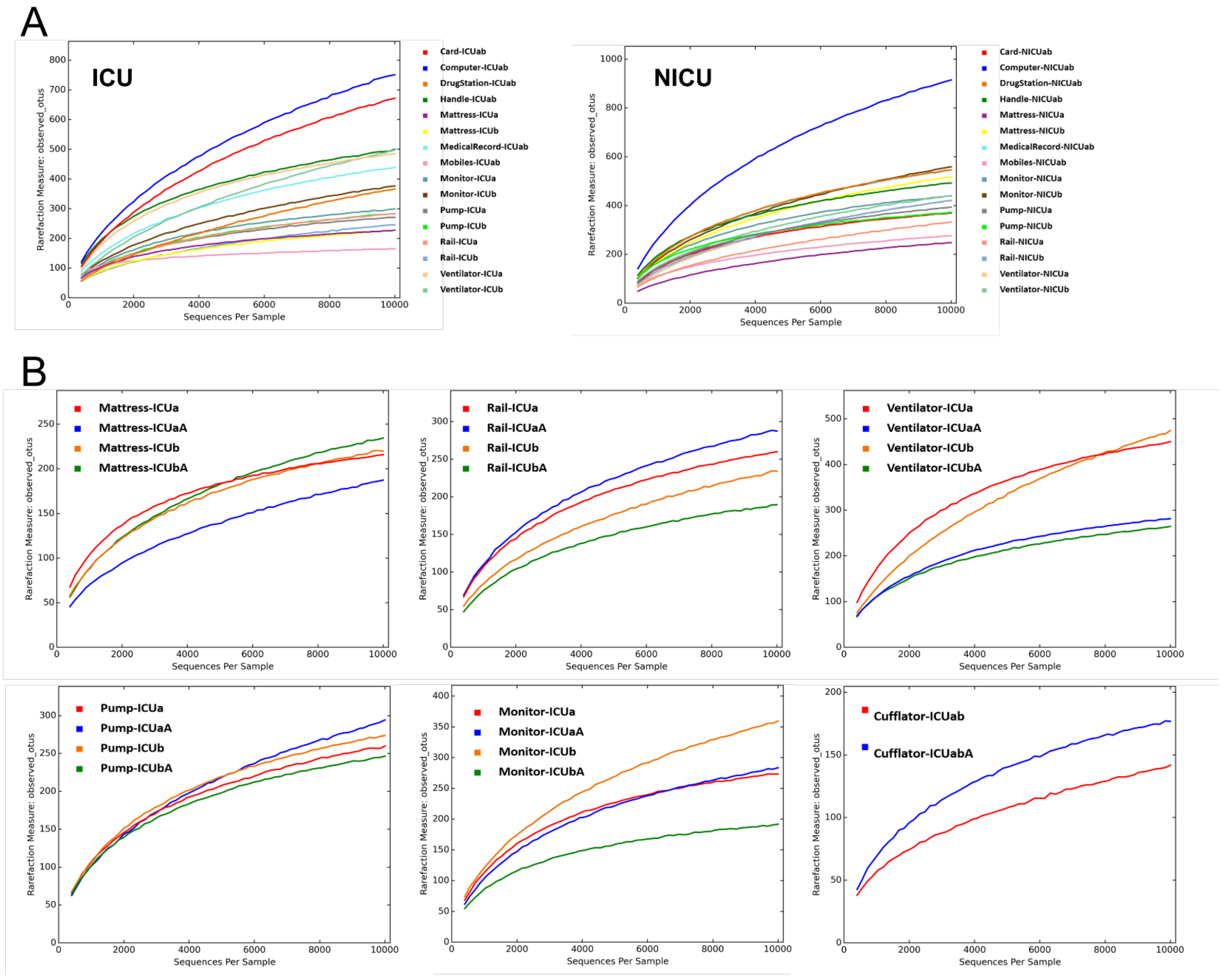

Figure S1. Rarefaction curve showing the relationship between the sequencing per sample and the number of OTUs that these reads represent. (A) ICU and NICU, and **(B)** ICU before and after cleaning. Sequences were rarefied with 33.708 read counts per sample.

Figure S2

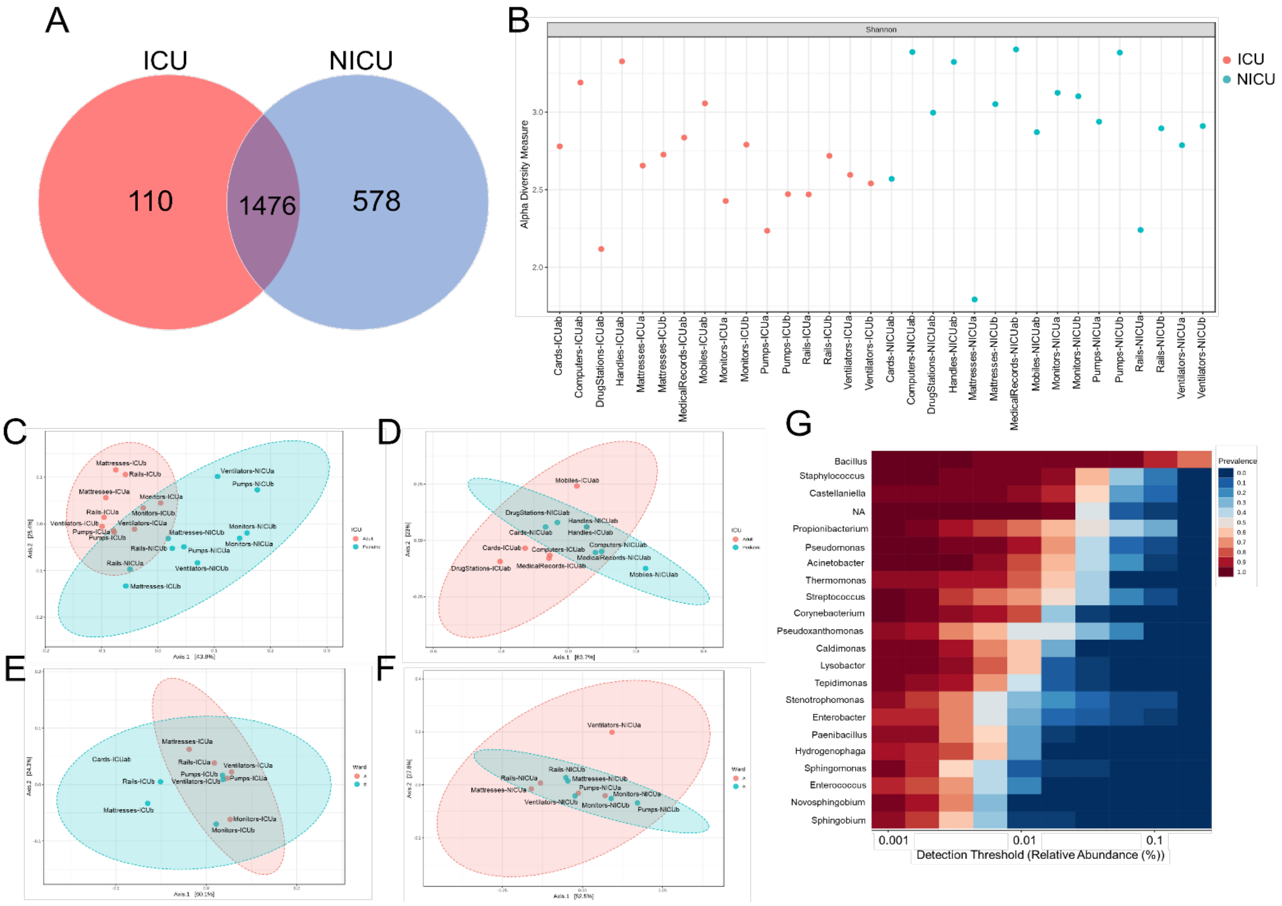

Figure S2. (**A**) Venn diagram showing shared and unique OTUs. (**B**) Alpha diversity at the OTU level for each sample at ICU (red) and NICU (cyan) calculated using Shannon index (Kruskal-Wallis test, p-value < 0.05). PCoA plot based on Jensen-Shannon distances between bacterial communities associated with (**C**) ICU and NICU boxes areas (ANOSIM, R= 0.50756; p-value < 0.001); (**D**) ICU and NICU common areas (ANOSIM, R= 0.14074; p-value = 0.116); (**E**) ICU wards (ANOSIM, R= 0.124; p-value = 0.177); (**F**) NICU wards (ANOSIM, R= -0.02; p-value = 0.52). Samples are shown as single dots. (**G**) Core microbiome analysis based on relative abundance and sample prevalence of bacterial genus in ICU and NICU. Divergence at OTU level was computed on Total sum scaling–normalized (TSS-normalized) datasets.

Figure S3

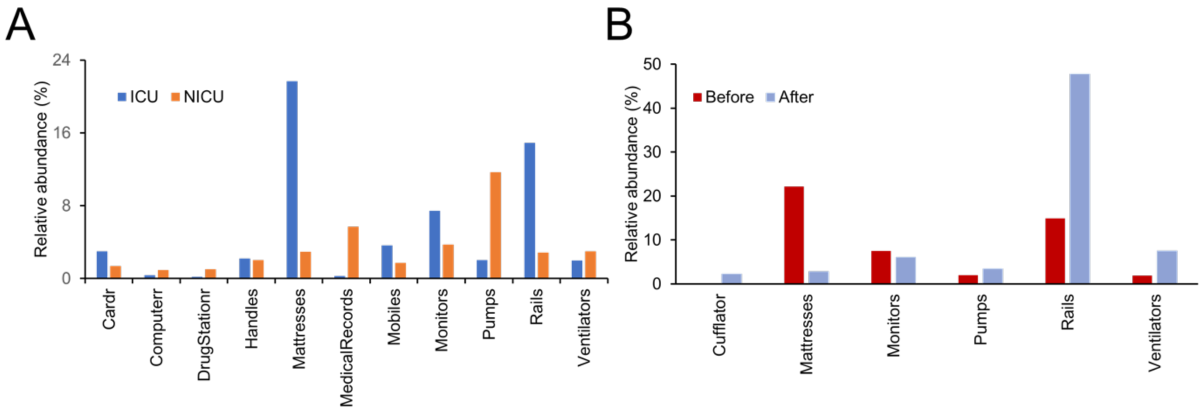

Figure S3. The abundance of fecal indicators in **(A)** ICU/NICU and **(B)** ICU before/after cleaning.

Figure S4

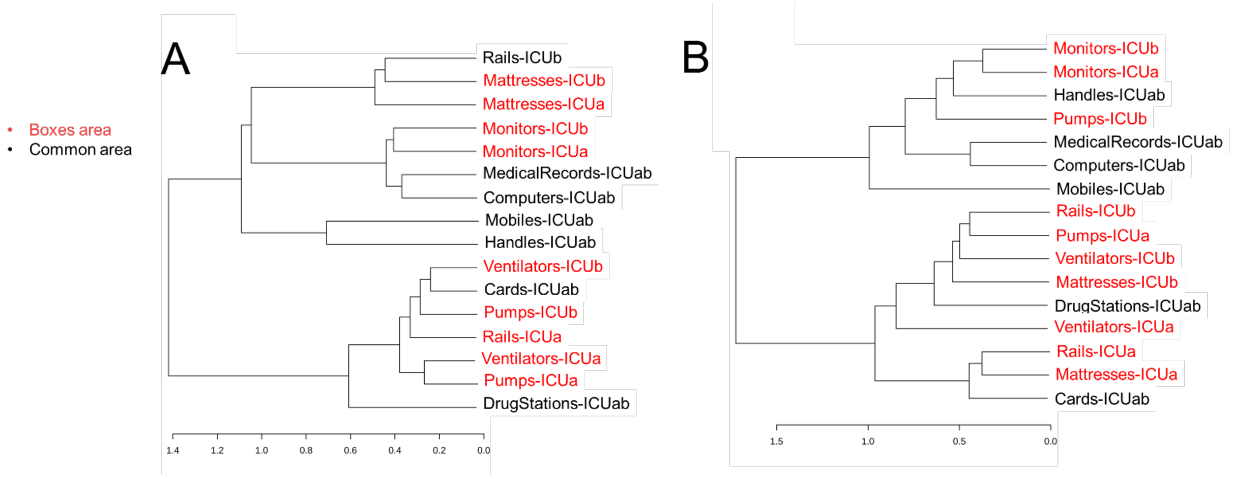

Figure S4. Dendrogram showing the similarities between samples. **(A)** ICU; **(B)** NICU. The dendrogram was created using the Jaccard index as distance measure and Ward's clustering algorithm.

Figure S5

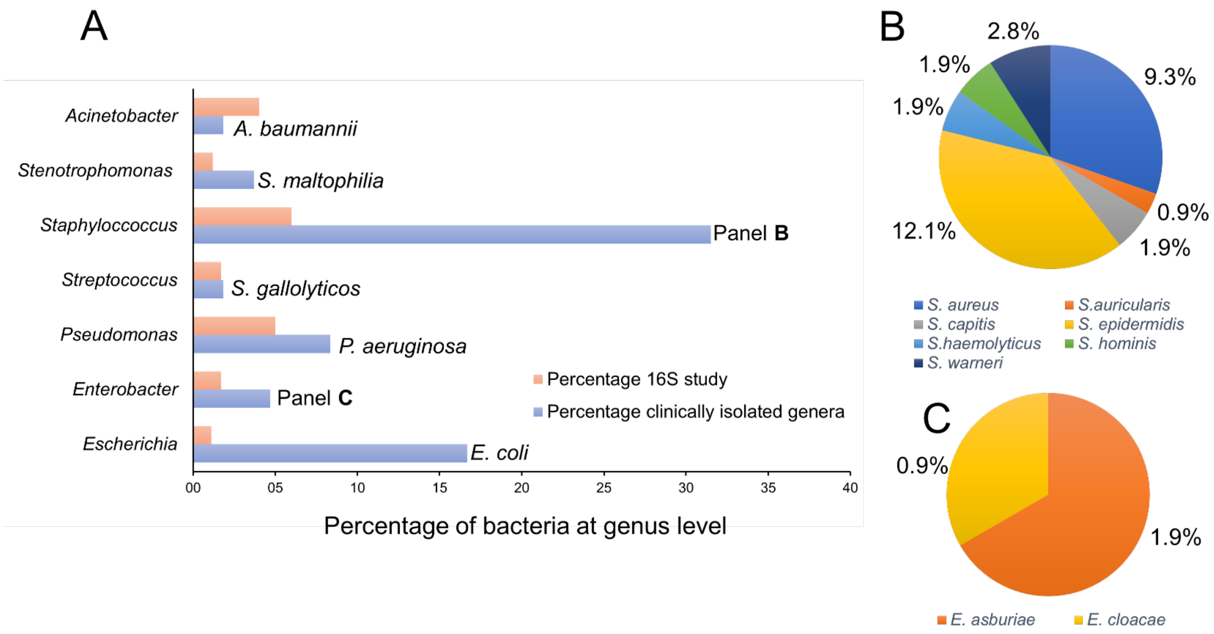

Figure S5. Percentage of identified bacteria at the genus level from the 16S analysis and isolated bacteria from clinical samples. (**A**) A total of 108 bacterial strains (gathered in 12 different genera) isolated from blood, bronchoalveolar lavage, peritoneal, cerebrospinal and ascitic fluids of hospitalized patients in the ICU were evaluated. In the graph are represented only the seven most abundant genera from the 16S amplicon study (considering up to 4% of all quality sequences). Data at the level of species are presented just for the bacteria isolated from clinical samples. Percentage of species belonging to the genera *Staphylococcus* (**B**) and *Enterobacter* (**C**), concerning the 108 bacterial strains isolated. For the other genera, single species were identified among biological samples, as presented in panel A.

Figure S6

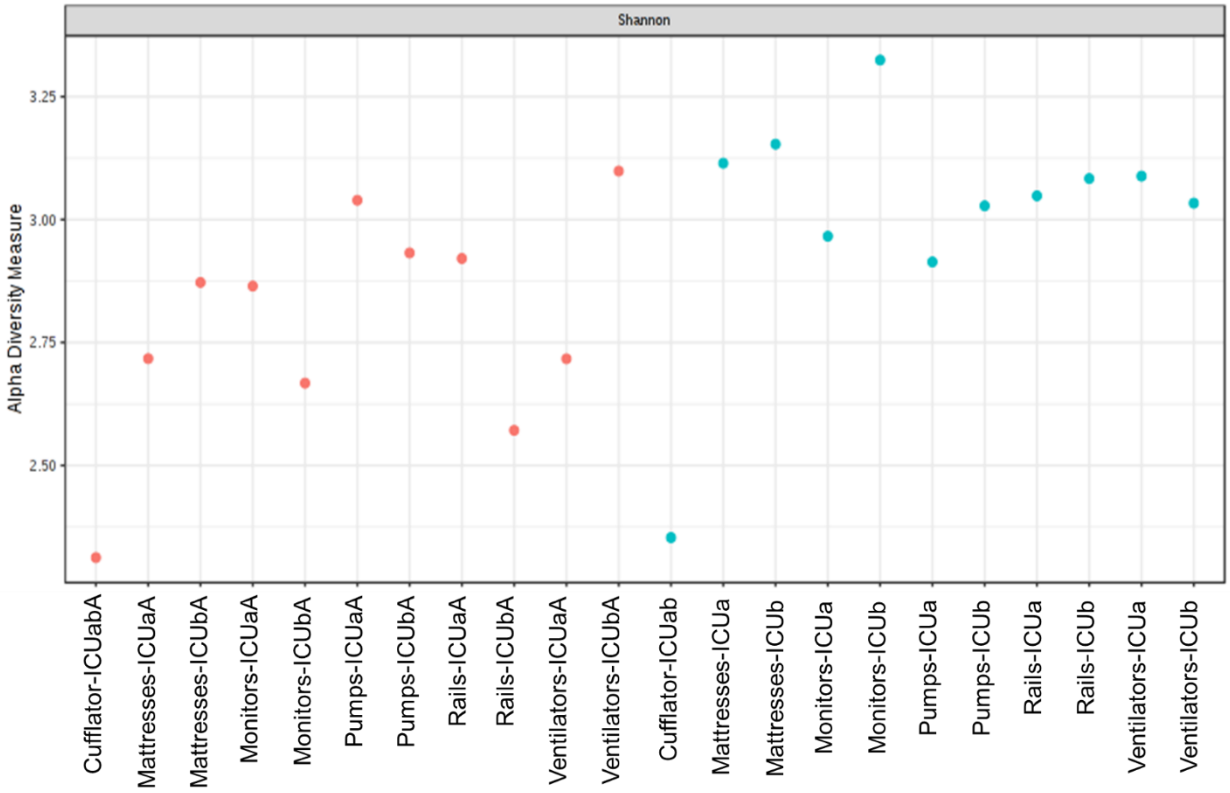

Figure S6. Alpha diversity at OTU level for each sample at ICU before (red) and after cleaning (cyan) calculated using Shannon index (Kruskal-Wallis test, p-value < 0.05).

Figure S7

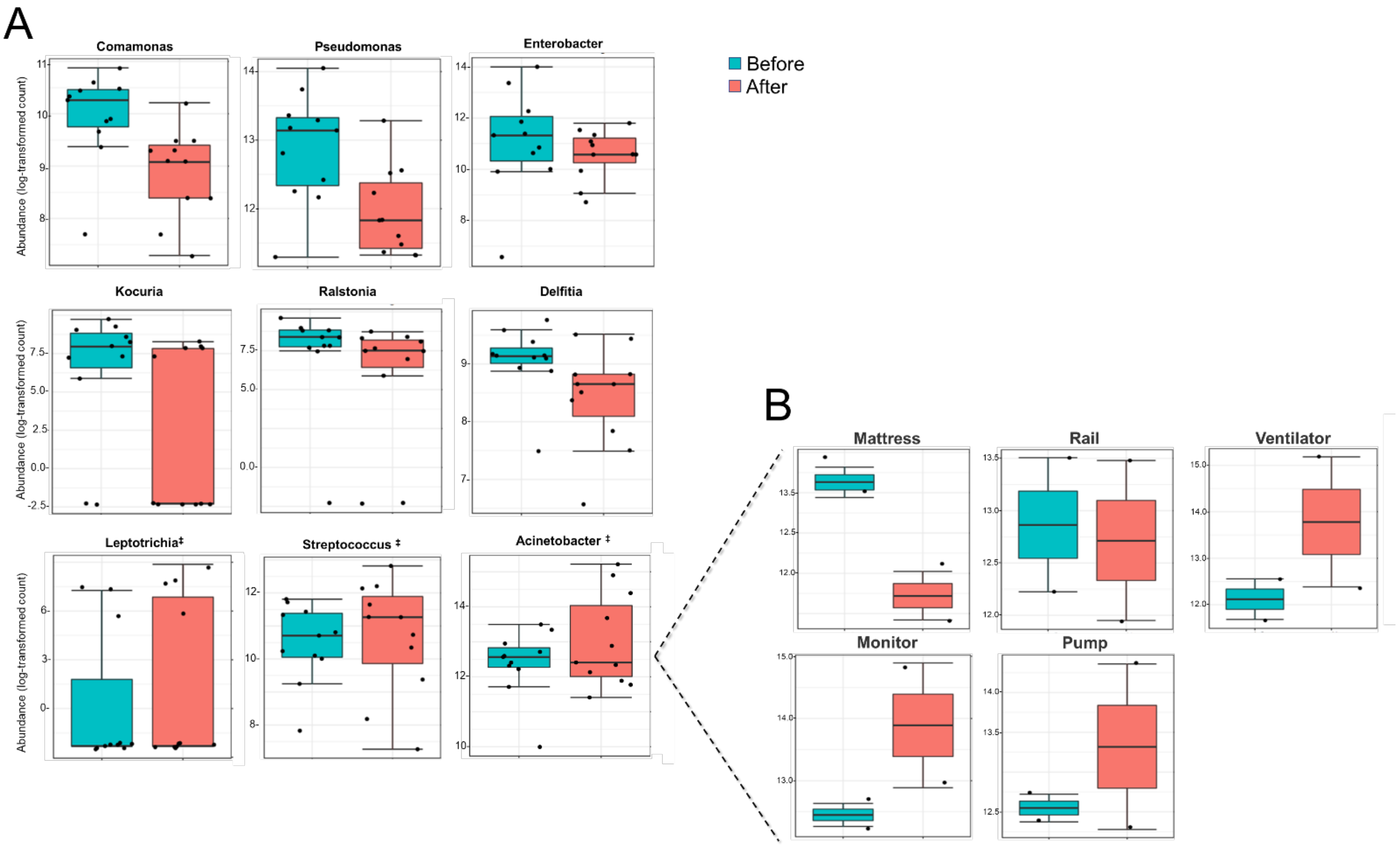

Figure S7. (**A**) Boxplot of relative abundance (log scale) of the genera HAI-related before (cyan) and after (red) cleaning. The difference was calculated using Mann-Whitney/Kruskal-Wallis test (FDR ≥0.3). ‡Genera with higher abundance after cleaning. (**B**) Differential relative abundance for *Acinetobacter* across all the ICU samples.

Supplementary text

Oxygen tolerance

Most of the samples contained a mixture of organisms with various degrees of oxygen tolerance. The number of strictly aerobic genera were highly represented (50%) followed by facultative anaerobe (36%) and obligatory anaerobic bacteria (14%) for both units. Infections caused by anaerobic bacteria are often underestimated, due to the difficulty to isolate and identify these microorganisms. The use of unspecific therapy against these infections may cause clinical failures [61]. Most abundant anaerobic organisms in ICU were, on decrescent order, *Propionibacterium*, *Bacteroides*, and *Prevotella*, whereas for NICU were *Propionibacterium*, *Prevotella*, and *Veillonella*. *Propionibacterium* is a human skin-associated genus [62], while *Prevotella* and *Veillonella* are part of the healthy microflora in the oral cavity and vaginal [51,63]. However, many species of *Prevotella*, and *Veillonella* genera are pathogens that cause oral or respiratory diseases[64], as well as meningitis [64]. *Veillonella* has also been involved in prosthetic cardiac valve or joint infections [65,66] and fatal sepsis [67]. *Bacteroides* species are usually part of the gastrointestinal microbiota [68], and they make a significant portion of the fecal bacterial population [69]. Among all anaerobic bacteria, *Bacteroides*, *Prevotella*, and *Veillonella* are the most frequently isolated in clinical samples of infection [70].

Gram-positive bacteria

Gram-positive bacteria were found in higher abundance in both units (Gram-positive and Gram-negative at ICU ─ 49% and 46%; NICU ─ 52% and 44.5%, respectively; ~5% were Gram-variable). Nonetheless, in terms of the number of genera, Gram-negative bacteria were predominant in both ICU (70%) and NICU (66%). Over the last decades, the focus of infection control centers was targeting Gram-positive pathogens due to their high rate of morbidity and mortality [71]. However, the incidence of infections in UTIs caused by Gram-negative bacteria has been rising alarmingly, requiring a better understanding of hospital microbiomes [72–74].

The five more abundant Gram-positive genera found in both ICU and NICU samples were, in order of decreasing abundance, *Bacillus*, *Staphylococcus*, *Propionibacterium*, *Streptococcus*, and

Corynebacterium. Core microbiome analysis was performed combining samples from both ICU and NICU. In total, 21 genera were shared in 80% of all samples at the minimum detection threshold of 0.001% relative abundance (**Fig. S2G**). The most abundant Gram-positive genera were also among the top 10 more prevalent. Most notably, *Bacillus* was the most prevalent genus in the core microbiome for both care units. *Bacillus* was also most abundant in ICU (36%) and NICU (26%) (**Fig. 2A**), mainly in the boxes area. To examine more deeply the bacterial community variations among the samples, a heatmap of the top 52 genera is illustrated in **Fig. 3A**. Accordingly, the ICU pumps and NICU mattresses contained the highest abundance of the total reads (~6%) (**Fig. 3A**). The identification of the *Bacillus* genus in hospital samples is often considered clinically safe since it is ubiquitous in the environment. However, recently outbreaks of severe and lethal *Bacillus* infections have been widely reported, especially diseases related with *Bacillus cereus* at NICUs [75,76]. Several of these infections resulted of contamination of respiratory equipment [76–78]. Therefore, contamination with this genus should not be routinely neglected.

Several clinical and metagenomic studies have described Gram-positive bacteria as a highly frequent colonizer of the skin [52,79]. The skin-associated genera *Staphylococcus*, *Propionibacterium*, *Streptococcus*, and *Corynebacterium*, were found in high abundance in surfaces frequently touched by hands of healthcare workers (HCW) such as, in order of decreasing abundance, computers, door handles, medical records, monitors, and mobiles (**Fig. 3A**). Most of the bacteria of these genera are harmless but may become opportunistic pathogens for immunocompromised patients [39]. Moreover, a high abundance of these genera was also observed in ventilators and pumps, suggesting that skin contact may be an essential source of contamination. *Staphylococcus* was found in all samples with a total abundance of 6% for both ICU and NICU. In the boxes area, this genus was more present on mattresses in ICU and ventilators in NICU (**Fig. 3A**). *Staphylococcus* can be found on the skin or in the nose of healthy patients causing no disease or only minor skin infections. However, several species can be deadly when invading bloodstream, joints, bones, lungs or heart [80]. *Staphylococcus aureus* is the most pathogenic and well-established species in the hospital environment.

Furthermore, coagulase-negative species such as *S. epidermidis*, *S. sciuri*, and *S. haemolyticus* are also an emerging problem in UTIs [80–82]. *Streptococcus* was found in all samples with a total abundance of 1.7% and 5% for ICU and NICU, respectively. In the boxes area, this genus was more present on monitors for both ICU and NICU (**Fig. 3Aa**). Nosocomial infections with *Streptococcus spp.* are often associated with respiratory or skin diseases [83] and cause long days of hospitalization

[84]. Species such as *S. pneumoniae* is the first most common cause of fatal bacterial pneumonia in developing countries with high morbidity in children [85]. Group B *Streptococcus*, a commensal bacterium, is the leading cause of death from early-onset infections in the neonate [86].

Other Gram-positive genera related to nosocomial infection were found but in intermediate abundance (0.5-1%), as, e.g., *Gemella*, *Enterococcus*, and *Clostridium*. These genera were present mainly in NICU being more abundant in ventilators (**Fig. 3Aa**). However, they were also found in pumps (*Enterococcus)*, door handles (*Clostridium*) and computers (*Gemella*). A very low abundance (≤0.1%) of these genera was observed in ICU samples, e.g., room cards).

Gram-negative bacteria

In the last decade, Gram-negative strains have gotten attention in their ability to spread their antibiotic resistance in hospital environments [87]. New molecular protocols, such as NGS, have allowed identifying emerging threats associated with nosocomial infections and multidrug-resistant [16]. An analysis involving eight ICUs reported that Gram-negative organisms were the principal responsible for HAI [88]. Here, the five more abundant Gram-negative genera found in ICU samples were, in order of decreasing abundance, *Pseudomonas*, *Pseudoxanthomonas*, *Castellaniella*, *Acinetobacter*, *Thermomonas*. Whereas for NICU were *Castellaniella*, *Delftia*, *Acinetobacter*, *Stenotrophomonas*, *Pseudomonas*. Previous studies have reported *Acinetobacter* and *Pseudomonas* as typical Gram-negative genera on (N)ICU surfaces [39].

Pseudomonas, *Acinetobacter*, *Delftia*, and *Stenotrophomonas* are known for their facultative pathogenic nature and for being nosocomial bacteria. *Pseudomonas* constituted 5%, and 2.6% of the bacterial community at ICU and NICU, respectively. The ICU mattresses and NICU drug station contained the highest abundance (**Fig. 3A**). *Acinetobacter* was found in all samples with a total abundance of 4% and 3% for ICU and NICU, respectively. *Acinetobacter* was more frequent on ICU mobiles. In the boxes area, this genus was more present on ICU mattresses and NICU monitors (**Fig. 3A**). *Delftia* showed an abundance of 1% at ICU and 5.5% at NICU, being more frequent on mobiles and NICU monitors (**Fig. 3A**). *Stenotrophomonas* showed an abundance of 1.2% and 2.7% for ICU and NICU, respectively. This genus was more present on NICU drug station and ventilators (**Fig. 3A**). *Enterobacter* and *Escherichia_Shigella* were also found in high abundance in both units, mainly in

pumps (NICU), and ICU bed matrasses and rails (**Fig. 3Aa**). In general, fecal indicators (*Enterobacter, Escherichia, Bacteroides, Anaerobiospirillum, and Parabacteroides*) were more frequent in bed matrasses and rails for ICU, and pumps for NICU (**Fig. S3Aa**).

Others Gram-negative HAI-related genera such as *Elizabethkingia* (bed rails), *Neisseria* (mobiles), *Haemophilus* (ventilators), *Leptotrichia* (mobiles), and *Serratia* (pumps) were primarily present only in NICU. *Prevotella* and *Sphingomonas* were found in both units in intermediate abundance.
